# Altered local and remote functional connectivity in mild Alzheimer’s disease patients with sleep disturbances

**DOI:** 10.3389/fnagi.2023.1269582

**Published:** 2023-10-18

**Authors:** Lei Wang, Rui Zhu, Xiao Zhou, Zhiyong Zhang, Dantao Peng

**Affiliations:** ^1^Department of Neurology, Beijing Geriatric Hospital, Beijing, China; ^2^Department of Neurology, The Second Hospital of Tianjin Medical University, Tianjin, China; ^3^Department of Neurology, China-Japan Friendship Hospital, Beijing, China

**Keywords:** Alzheimer’s disease, sleep disturbances, rs-fMRI, functional connectivity, ReHo

## Abstract

**Objectives:**

This study aimed to investigate local and remote functional connectivity in mild Alzheimer’s disease patients with sleep disturbances (ADSD) and those without sleep disturbances (ADNSD).

**Methods:**

Thirty eight mild AD patients with sleep disturbances and 21 mild AD patients without sleep disturbances participated in this study. All subjects underwent neuropsychological assessments and 3.0 Tesla magnetic resonance scanning. Static and dynamic regional homogeneity (ReHo) were used to represent the local functional connectivity. Seed-based whole-brain functional connectivity was used to represent the remote functional connectivity. The seed was chosen based on the results of ReHo.

**Results:**

Compared to ADNSD, ADSD showed decreased static ReHo in the left posterior central gyrus and the right cuneus and increased dynamic ReHo in the left posterior central gyrus. As for the remote functional connectivity, comparing ADSD to ADNSD, it was found that there was a decreased functional connection between the left posterior central gyrus and the left cuneus as well as the left calcarine.

**Conclusion:**

The current study demonstrated that, compared with ADNSD, ADSD is impaired in both local and remote functional connectivity, manifested as reduced functional connectivity involving the primary sensory network and the primary visual network. The abnormality of the above functional connectivity is one of the reasons why sleep disorders promote cognitive impairment in AD. Moreover, sleep disorders change the temporal sequence of AD pathological damage to brain functional networks, but more evidence is needed to support this conclusion.

## 1. Introduction

The most prevalent progressive neurodegenerative disease, Alzheimer’s disease (AD), is characterized by continuous progressive cognitive dysfunction and impairment of mental conduct. Memory loss, language and visuospatial impairment, disorders of abstract thought and computation, and personality and behavior problems are examples of clinical manifestations (Alzheimer's Association, 2016). As society ages, its prevalence rises annually, placing a significant psychological and financial strain on AD patients, caregivers, and society as a whole (Wong, 2020).

Alzheimer’s disease cognitive impairment is caused by extracellular beta-amyloid (Aβ) plaque and intracellular neurofibrillary tangles containing aggregated microtubule-related tau, as well as synaptic dysfunction and loss (Forner et al., 2017). Both antemortem cognitive status and synapse loss in various brain regions tend to correspond with the severity of involvement by those two pathological hallmarks (Scheff et al., 2014). The oligomeric form of Aβ induces synaptic loss *in vitro* and *in vivo*, suggesting that Aβ is a driver of AD synaptic dysfunction (Mucke and Selkoe, 2012; Ittner and Ittner, 2018). Aβ-induced synaptic loss leads to neural network dysfunction in patients with AD, which is manifested as hypersynchronous or epileptic discharge in electroencephalogram recordings (Palop and Mucke, 2010). Similarly, synaptic loss and neural network abnormalities occur in transgenic mouse models of human amyloid precursors (Verret et al., 2012). Aβ-induced synaptic and neural network defects are closely related to tau, as reducing endogenous tau ameliorates Aβ-induced deficits in an AD mouse model mediated by postsynaptic molecular changes (Roberson et al., 2007). Conversely, overexpression of human wild-type tau in a mouse model results in the lasting changes in dendritic spines and cognitive impairment found in AD (Chabrier et al., 2014). Meanwhile, there is a robust correlation between synapse numbers and cognitive capacity (Scheff et al., 2011). Warren et al. proposed the term “molecular nexopathy” to define the network signatures of neurodegenerative pathologies that result from the coherent conjunction of pathogenic proteins and intrinsic neural network features. In the molecular nexopathy paradigm, networks show variable intrinsic vulnerability to proteinopathies (including AD, frontotemporal lobar degeneration, and corticobasal degeneration) (Warren et al., 2013). Likewise, another study suggested a specific pattern of network degradation associated with the spreading of AD pathology within targeted neural networks (Chhatwal, et al., 2018). For example, Aβ fibrils start to accumulate predominantly within certain parts of the default-mode network in preclinical AD and already affect brain connectivity (Palmqvist et al., 2017). According to these findings, AD is primarily a disconnection syndrome (Delbeuck et al., 2003). Thus, synaptic dysfunction or loss, together with the ensuing weakening of neuronal connections, appear to be ongoing effects of AD pathology and contribute to the ongoing decline in cognitive function in AD patients.

Sleep disorders are widespread in AD patients, which may precede cognitive symptoms and exacerbate cognitive decline (Li et al., 2019). Studies have confirmed that sleep disorders promote the development of AD pathology, including Aβ and tau (Wang and Holtzman, 2020). Underlying physiological changes of soluble Aβ and tau in the brain interstitial fluids are regulated by a circadian rhythm of synaptic/neuronal activity, increasing during wakefulness and decreasing during sleep. Aβ and tau are among the metabolites that are eliminated by the glymphatic system through the exchange of cerebrospinal fluid and interstitial fluid. Sleep is when the glymphatic system’s purge function is most active. In accordance with the effectiveness of metabolite clearance, the exchange of perivascular cerebrospinal fluid and interstitial fluid during sleep is substantially higher than it is during waking. In AD patients, sleep disorders can cause extended sleep latency, heightened nighttime awakenings, and reduced duration of non-rapid eye movement sleep (especially slow-wave sleep), which prolongs nocturnal awakenings. Eventually, this can lead to an increase in the production or release of Aβ and tau by synapses/neurons, and a decline in their clearance within the glymphatic system. Sleep disorders can promote tau phosphorylation and proliferation (Ju et al., 2014; Boespflug and Iliff, 2018; Harrison et al., 2020; Wang and Holtzman, 2020). Sleep deprivation can worsen memory loss and synaptic loss, as well as harm synaptic integrity in AD mouse models, according to animal studies (Di Meco et al., 2014; Kincheski et al., 2017). Tononi and others have suggested in notable studies that sleep deprivation impacts cognition, including learning ability, memory consolidation and integration, and behavioral performance (Tononi and Cirelli, 2014; Boly et al., 2017; Nir et al., 2017). As a result, sleep disruption may increase synapse dysfunction and loss as well as cognitive decline by encouraging the pathological emergence of AD.

Resting-state functional MRI (rs-fMRI) is a promising neuroimaging technique that can non-invasively measure spontaneous or intrinsic brain activity (IBA) (Biswal et al., 1995). It has been extensively utilized in research on functional relationships in populations with disease and health, especially AD (Fox and Raichle, 2007). An investigation of the intrinsic functional connectivity patterns of the whole-brain neural network in AD was conducted utilizing rs-fMRI and voxel-based graph theory analysis. It showed that functional damage to the hubs in the brain’s neural network has a distance dependence feature, with long-distance connections suffering the most serious damage. AD patients have impaired functional connections within the executive control network, salience network, default mode network, and between the executive control network and salience network when compared to healthy controls (Dai et al., 2015). A study used regional homogeneity (ReHo) based on two-dimensional cortical surfaces to represent local functional connections and whole brain functional connections based on seeds to represent remote connections. Similarly, it was found that functional connectivity damage is correlated with distance in patients with amnestic mild cognitive impairment (aMCI) (Zhang et al., 2017). These findings shed light on the pathophysiological underpinnings of AD as a connection disorder. In the rs-fMRI community, ReHo and seed-based functional connectivity are frequently utilized to describe local and remote cortical connections, respectively (Zhang et al., 2017). For measuring functional connectivity from BOLD fMRI data, two extensively utilized approaches are spatial independent component analysis (ICA) and temporal correlation with a designated seed voxel or small region of interest (ROI). In general, the outcomes of the seed-based and ICA techniques are comparable. The sum of ICA-derived within-network and between-network connectivities is demonstrated to be the formula for seed-based FC measurements (Joel et al., 2011). The first technique used to detect resting state networks was seed-based functional connectivity, which also gave a direct way to investigate the brain regions with robust functional connectivity using the seed (Cole et al., 2010; Smitha et al., 2017). ReHo has been demonstrated to have high robustness against temporal and spatial noise and outliers (Zuo et al., 2013). To our best knowledge, there has been no research on local and remote functional connectivity in AD associated with sleep disturbances.

An rs-fMRI study used ICA to reconstruct the frontoparietal network and default mode network in MCI patients with high and low self-reported sleep quality. It was found that high quality of sleep was associated with increased frontoparietal network connectivity among patients with MCI, and the increased connectivity may underlie compensatory mechanisms to overcome advancing neurodegeneration (Pini et al., 2020). In contrast to patients with MCI but no sleep disturbance, another study sought to identify abnormalities in default mode network functional connectivity (an ROI-to-ROI analysis) in patients with MCI and sleep disturbance. Participants with sleep disturbance in MCIs showed considerably less DMN connection between the temporal and parietal areas than those with normal sleep patterns. These brain areas underpin salient memory and sleep systems (McKinnon et al., 2016). However, in a previous study, we investigated the IBA intensity in mild AD patients with or without sleep disturbances using percent amplitude fluctuation and standardized percent amplitude fluctuation. It was found that the brain regions where IBA changes occurred were related to sleep regulation and mainly located in the primary somatosensory and motor cortex (central anterior gyrus and central posterior gyrus) and the primary visual cortex (cuneus) (Wang and Peng, 2021). These areas are mainly affected in the later stages of AD pathology (Thal et al., 2002; Braak et al., 2006). This means that AD patients with sleep disorders may have a different functional connectivity damage pattern than those with pure AD.

In this study, we used ReHo and seed-based whole-brain functional connectivity to compare the local and remote functional connectivity between mild AD patients with sleep disturbances (ADSD) and mild AD patients without sleep disturbances (ADNSD). We propose that functional connectivity in particular brain areas, traditionally associated with sleep regulation, differs in ADSD from ADNSD. These distinctive alterations could contribute to a better understanding of how sleep disturbances impact brain function in AD patients.

## 2. Materials and methods

### 2.1. Subjects

From the Outpatient Department of Neurology at the China-Japan Friendship Hospital, 41 mild AD patients with sleep disorders and 28 mild AD patients without sleep disorders participated in the current study. Each patient was right-handed (examined by the Edinburgh Inventory handedness test) and had a set caregiver. An experienced neurologist in the field of cognitive dysfunction established the conclusive diagnosis of probable AD dementia in accordance with the National Institute of Aging-Alzheimer’s (NIA-AA) criteria from 2011 (McKhann et al., 2011). The Chinese version of the Pittsburg sleep quality index (PSQI) was applied for grouping with a cutoff value of 8 (Qiu et al., 2020). The participants were in the mild dementia stage, as confirmed by their clinical dementia rating score of 1. To be noted, patients in the ADSD group developed sleep disorders after displaying cognitive symptoms. Sleep disorders lasted for at least 1 month or up to 3 months. Prior to any medical intervention for sleep disorders, a series of evaluations were conducted over a period of 4 days, including medical history collection, neurological examination, neuropsychological test, and rs-fMRI.

The exclusion criteria were specified as follows: (a) Hachinski Ischemic Score (13 items) >4; (b) Hamilton Depression Scale (17 items) >10; (c) other neurological diseases or systemic diseases that could induce cognitive dysfunction (e.g., vascular dementia, dementia with Lewy Body, frontotemporal dementia, epilepsy, encephalitis, brain tumors, traumatic brain injury, Parkinson’s disease, thyroid dysfunction, syphilis, HIV, Vitamin B12 deficiency, alcohol abuse, or drug abuse); (d) a history of mental disorders; (e) major or severe medical diseases (e.g., cancer, uncontrolled diabetes mellitus, severe heart failure, acute infection; severe cervical or cerebral vascular stenosis); (f) those who were unable to complete the neuropsychological tests or MRI; or (g) those with head motion exceeding >3 mm or 3 degrees. This research was undertaken with the authority of the China-Japan Friendship Hospital Ethics Committee (2015-GZR-16). Participants provided written informed consent prior to taking part in the study.

### 2.2. MRI data acquisition

MRI data was collected using a 3.0 Tesla MRI scanner (GE Discovery MR750, Milwaukee, United States) equipped with a standard head coil. Participants were instructed to keep their eyes closed, remain still, and not fall asleep during the data collection process. After the resting-state scans, all subjects were asked to verify that they remained awake throughout the scan. This study excluded participants who were unable to provide feedback or displayed signs of falling asleep. Meanwhile, the 8 min rs-fMRI was performed first, followed by the structural MRI. The rs-fMRI was obtained using a single-shot gradient-recalled echo-planar imaging sequence: repetition time (TR) = 2000 ms, echo time (TE) = 30 ms, flip angle (FA) = 90°, field of view (FOV) = 224 mm × 224 mm, matrix = 64 × 64, number of excitations (NEX) = 1, slice spacing = 0.7 mm, slice thickness = 3.5 mm, 34 slices and 240 time points. 3D-T1WI anatomic images were acquired using three-dimensional fast spoiled gradient-echo sequences (3D FSPGR): slice thickness = 1.0 mm, TR = 6.7 ms, TE = Min Full, acquisition matrix = 256 × 256, FOV = 256 mm × 256 mm, NEX = 1.

### 2.3. Image preprocessing

Data Processing Assistant for Resting-State fMRI (DPABI V4.3) (Yan et al., 2016) and SPM8 (Statistical Parametric Mapping^1^) were used to preprocess imaging data based on the Matrix Laboratory software platform (2014a). The preprocessing steps included: (1) removing the first 10 time points; (2) slice timing correction with the following parameters: slice number = 34, slice order = [1:2:33, 2:2:34], and reference slice = 33; (3) Realign. The study employed a relatively lenient criterion for excluding participants with significant head motion (greater than 3 mm or 3 degrees) among AD patients; (4) Spatial normalization with 3D-T1WI by using new segment and Diffeomorphic Anatomical Registration Through Exponentiated Lie Algebra (DARTEL); (5) Nuisance covariates regression, including the linear trend of the time series, the Friston-24 head motion parameters, the white matter signals, and the cerebrospinal fluid signals; (6) Spatial smoothing with a 4 mm full width at half maximum (FWHM) Gaussian kernel (not for ReHo); and (7) band-pass (0.01–0.10 Hz) filtering.

### 2.4. rs-fMRI parameters calculations

#### 2.4.1. Static regional homogeneity

Static ReHo is a data-driven method for analyzing rs-fMRI data that aims to evaluate the regional brain area’s synchronization of IBA, thereby reflecting the local functional connectivity (Zhang et al., 2017). Kendall’s coefficient concordance (KCC) is obtained by calculating the similarity between the time series of a given voxel and its nearest voxel (26 voxels in this study), which represents the ReHo value of that particular voxel (Zang et al., 2004). A higher ReHo value indicates a stronger temporal coherence in the BOLD signal among neighboring voxels (Zhang et al., 2017). DPABI V4.3 was utilized in this study to compute the ReHo value of each voxel within the brain, which was subsequently standardized by dividing it with the mean value of all voxels’ ReHo across the entire brain to obtain mReHo. The resulting mReHo maps were then smoothed with a Gaussian kernel of 4 mm FWHM to generate smReHo for statistical analysis.

#### 2.4.2. Dynamic regional homogeneity

Unlike static ReHo, which assumes the BOLD signal remains stable throughout the rs-fMRI scan, dynamic ReHo focuses on the temporal dynamics of IBA. It employs the sliding window approach to effectively capture the dynamic changes of local IBA in the temporal domain (Chen et al., 2018). The sliding window method divides the entire scanning period into multiple windows and independently calculates the resting state functional index in each window (Allen et al., 2014). This method involves two crucial parameters: window length and step size. The selection of the appropriate window length is critical, as it must be small enough to detect potential transient signals while also being large enough to describe the lowest frequency of interest in the signals (Sakoğlu et al., 2010). Previous research has shown that in order to eliminate spurious oscillations, the minimum window length should be greater than 1/fmin (where fmin is the minimum frequency of the time series) (Leonardi and Van De Ville, 2015). A step size of 1TR, 2TR, and 5TR was chosen based on prior studies (Yu et al., 2019). Therefore, we estimated the dynamic ReHo under four conditions to avoid the effects of window length and step size (window length = 50 TR, 60 TR, 60 TR and 75 TR; step size = 1 TR, 2 TR, 5 TR and 2 TR correspondingly; TR = 2 s). The dynamic ReHo calculation in the current study utilized Temporal Dynamic Analysis (TDA) toolkits based on RESTplus V1.25 (Jia et al., 2019). By calculating the ReHo of all voxels in time windows, each participant got a series of window-based ReHo maps. The next step was to determine the mean and standard deviation of each voxel across all of the window-based ReHo maps for each participant. The standard deviation was then divided by the mean to determine the corresponding coefficient of variation (CV). On behalf of dynamic ReHo, CV was lastly used for statistical analysis.

#### 2.4.3. Seed-based functional connectivity

The functional connectivity analysis was performed using DPABI V4.3, and brain regions with statistically significant differences in static ReHo and dynamic ReHo were selected as seeds. A correlation map was produced for each participant by extracting the BOLD time series from each seed and computing Pearson’s correlation coefficients between the time series in every seed and all other voxels of the entire brain. Correlation coefficients were Fisher transformed into ‘Z’ scores to increase normality for statistical analysis.

### 2.5. Statistics analysis

The demographic and clinical data between the ADSD and ADNSD groups were compared using the two-sample *t*-test or Mann–Whitney *U* test and the Chi-square test with SPSS 22.0 (IBM Corp., Armonk, NY). All tests were double-tailed, and *p* < 0.05 was considered statistically significant.

To examine the between-group differences in the three measurements, a two-sample *t*-test was held between the ADSD and ADNSD groups using RESTplus V1.25 with age, gender, head motion, education year, and gray matter density as covariates. Multiple comparison correction was performed based on Gaussian random field theory (GRF, voxel-wise *p* < 0.001, cluster-wise *p* < 0.05, two-tailed).

## 3. Results

### 3.1. Demographic and clinical data

There were 38 participants in ADSD and 21 participants in ADNSD after excluding those whose head motion exceeded 3 mm or 3 degrees (3 participants in ADSD and 7 participants in ADNSD). No significant differences were found (*p* > 0.05) in the demographic and clinical characteristics between the ADSD group and the ADNSD group, except for the PSQI score (*p* < 0.05) (Tables 1, 2).

**Table 1:** Demographic and clinical data.

	ADSD (*n* = 38)	ADNSD (*n* = 21)	U/χ^2^	*p*
Age(year)	73.7 ± 7.2	73.6 ± 8.4	392.5^b^	0.918
Gender(M/F)	12/26	8/13	0.256^a^	0.613
Education(year)	12.0 ± 3.9	12.6 ± 4.3	353.5^b^	0.455
Head motion*	0.156 ± 0.084	0.183 ± 0.101	333.0^b^	0.296
ApoE4, *n* (%)	16 (42.1%)	10 (47.6%)	0.167^a^	0.683
HT, *n* (%)	17 (44.7%)	8 (38.1%)	0.244^a^	0.621
HP, *n* (%)	24 (63.2%)	14 (66.7%)	0.073^a^	0.788
LI, *n* (%)	31 (81.6%)	15 (71.4%)	0.382^a^	0.567
WMH (Fazekas Score), *n* (%)				
0	6 (15.8%)	2 (9.5%)	313.5^b^	0.126
1	21 (55.3%)	13 (61.9%)		
2	11 (28.9%)	6 (28.6%)		
MMSE	22.4 ± 1.7	22.9 ± 1.7	332.5^b^	0.285
CDR	1	1	-	-
HIS	1.1 ± 0.94	1.6 ± 0.93	286.5^b^	0.62
HAMD	2.37 ± 0.63	2.43 ± 0.51	367.5^b^	0.561
Adas-Cog	15.36 ± 3.38	13.82 ± 2.98	302.0^b^	0.124

ADSD = AD patients with sleep disturbances; ADNSD = AD patients without sleep disturbances; HT, Hypertension; HP, Hyperlipidemia; LI, Lacunar infarction; WMH, White matter hyperintensities; MMSE, Mini-Mental State Examination; CDR, Clinical Dementia Rating; HIS, Hachinski Ischemic Score (13 items); HAMD, Hamilton Depression Scale (17 items); Adas-Cog, Alzheimer’s Disease Assessment Scale-Cognitive section; ^a^ = χ2 value obtained by the Chi-Square test; ^b^ = U value obtained by the Mann–Whitney *U* test; * = mean frame displacement (FD) Jenkinson to represent the head motion.

**Table 2:** PSQI score.

	ADSD (*n* = 38)			ADNSD (*n* = 21)			*U*	*ρ*
PSQI Components	Median	Per25-75%	Mode	Median	Per25-75%	Mode		
SQ	2	2–2	2	1	1–1	1	63.0	0.000
SL	1	1–2	1	1	0–1	1	168.0	0.000
SD^1^	1	1–2	1	0	0–1	0	87.5	0.000
SE	1	1–2	1/2^a^	1	0–1	1	131.0	0.000
SD^2^	1	1–2	1	1	1–1	1	229.0	0.001
SM	NA							
DD	1	1–2	1	1	0–1	1	172.5	0.000
TS	9	8–10	8	4	3–5	5	0	0.000

ADSD = AD patients with sleep disturbances; ADNSD = AD patients without sleep disturbances; ^a^, Multiple modes exist (1 and 2); NA, Not applicable (PSQI was conducted before sleep medication); SQ, Sleep Quality; SL, Sleep Latency; SD^1^, Sleep Duration; SE, Sleep Efficiency; SD^2^, Sleep Disturbances; SM, Sleep Medication; DD, Daytime Dysfunction; TS, Total Score of PSQI.

### 3.2. Static ReHo and dynamic ReHo

**Figure 1:**
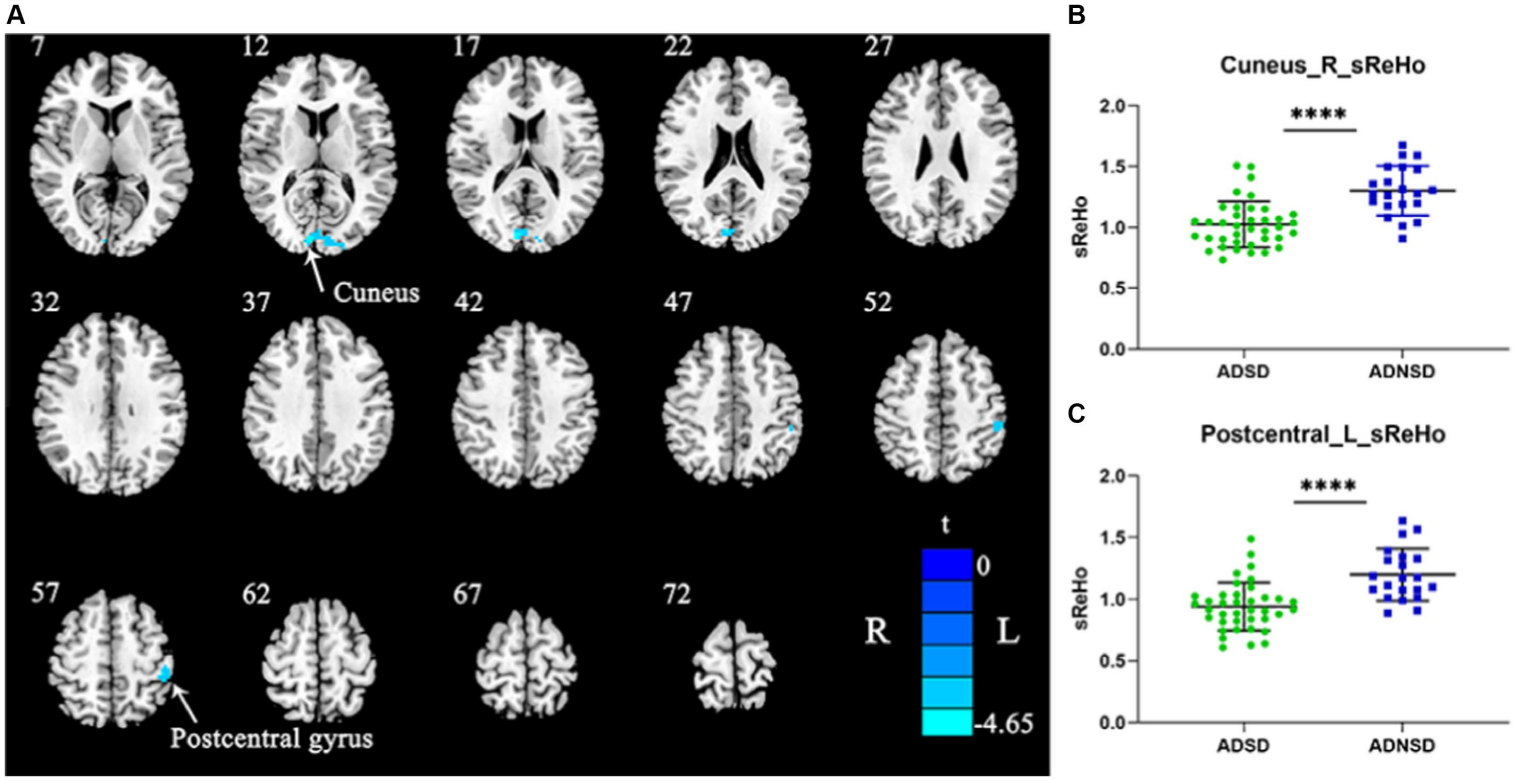
**(A)** Blue represents decreased static ReHo in the right cuneus and left posterior central gyrus in ADSD compared to ADNSD (two-sample t-test, GRF corrected, voxel-wise *p* < 0.001, cluster-wise *p* < 0.05, two-tailed). R = right hemisphere; L = left hemisphere; the color bar represents the *t* value. **(B,C)** show the difference between ADSD and ADNSD in static ReHo in the right cuneus and left posterior central gyrus. **** represents *p* < 0.0001. ADSD = AD patients with sleep disturbances; ADNSD = AD patients without sleep disturbances.

Compared to ADNSD, ADSD showed decreased static ReHo in the left posterior central gyrus and the right cuneus (Figure 1 and Table 3).

**Table 3:** Brain regions showing differences between ADSD and ADNSD.

Brain regions	BA	Cluster size (no. voxels)	Peak MNI coordinates			*t* value
			X	Y	Z	
Decreased static ReHo in ADSD						
Right cuneus	18	73	-9	−90	15	−4.65
Left posterior central gyrus	40	40	−45	−33	54	−4.56
Increased dynamic ReHo in ADSD						
Left posterior central gyrus	40	44	−39	−42	57	4.29
Decreased functional connectivity in ADSD (the left posterior central gyrus as seed)						
Left calcarine	17	55	0	−93	9	−4.62
Left cuneus	19	45	−6	−87	33	−4.67

ADSD = AD patients with sleep disturbances; MNI, Montreal Neurological Institute; BA, Brodmann area.

**Figure 2:**
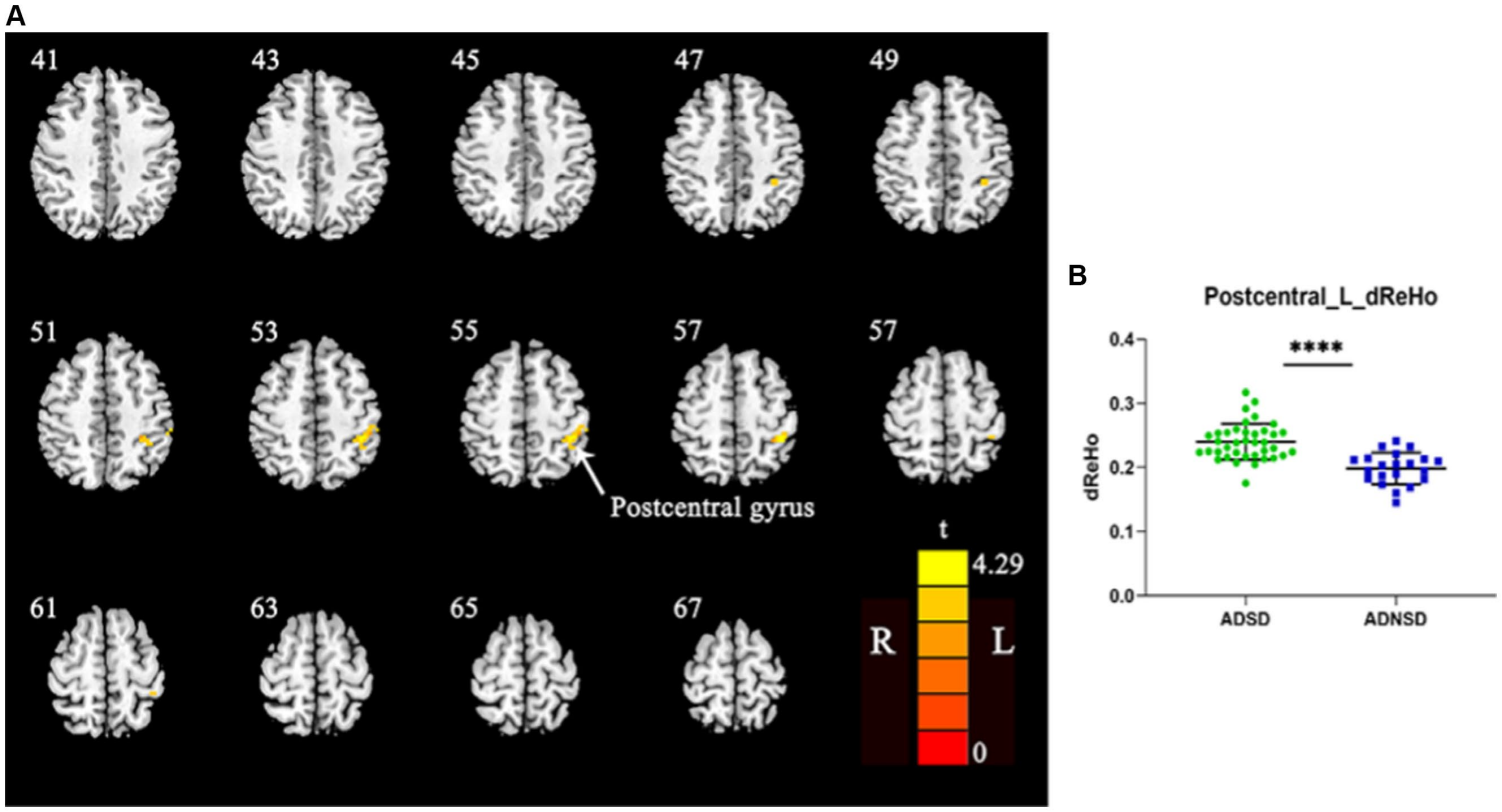
**(A)** Yellow represents increased dynamic ReHo in the left posterior central gyrus in ADSD compared to ADNSD (window width = 50TR, step size = 1TR; two-sample *t*-test, GRF corrected, voxel-wise *p* < 0.005, cluster-wise *p* < 0.05, two-tailed). R = right hemisphere; L = left hemisphere; The color bar represents the *t* value. **(B)** shows the difference between ADSD and ADNSD in dynamic ReHo in the left posterior central gyrus. **** represents *p* < 0.0001. ADSD = AD patients with sleep disturbances; ADNSD = AD patients without sleep disturbances.

There were no significant differences in dynamic ReHo between the ADSD and ADNSD when the threshold of the GRF was set as voxel-wise *p* < 0.001, cluster-wise *p* < 0.05, and two-tailed. A study by Jia et al. in 2021 suggested that small *p* values may not yield robust findings (Jia et al., 2021). Therefore, when the GRF threshold was adjusted to voxel-wise *p* < 0.005, cluster-wise *p* < 0.05, and two-tailed, ADSD showed increased dynamic ReHo in the left posterior central gyrus relative to the ADNSD in the aforementioned four conditions (Figure 2; Table 3, and Supplementary material).

### 3.3. Seed-based functional connectivity

**Figure 3:**
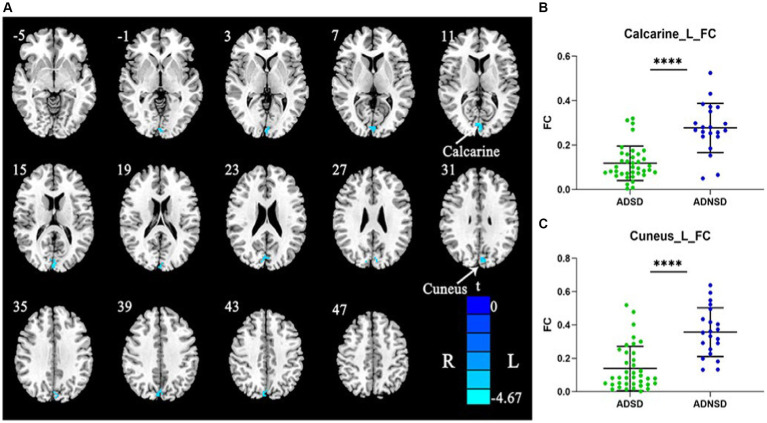
**(A)** Blue represents decreased remote functional connectivity between the left posterior central gyrus and the left cuneus as well as left calcarine in ADSD compared to ADNSD (the left posterior central gyrus as seed; two-sample t-test, GRF corrected, voxel-wise *p* < 0.001, cluster-wise *p* < 0.05, two-tailed). R = right hemisphere; L = left hemisphere; the color bar represents the *t* value. **(B,C)** show the difference between ADSD and ADNSD in remote functional connectivity in the left posterior central gyrus and the left cuneus as well as left calcarine. **** represents *p* < 0.0001. ADSD = AD patients with sleep disturbances; ADNSD = AD patients without sleep disturbances.

The left posterior central gyrus was chosen as the seed for whole-brain functional connectivity based on the findings of static and dynamic ReHo. Comparing ADSD to ADNSD, it was found that there was a decreased functional connection between the left posterior central gyrus and the left cuneus as well as the left calcarine (Figure 3 and Table 3).

## 4. Discussion

In the present study, we used ReHo and seed-based whole-brain functional connectivity to explore local and remote functional connectivity in ADSD and ADNSD. The results showed that compared to ADNSD, ADSD had impaired local and remote functional connectivity. The above-mentioned brain regions are related to sleep and cognitive functions and parts of the primary sensory cortex, which is consistent with our earlier research and raises the possibility that ADSD may have a particular pattern of functional connectivity impairment. No differences between the two groups were seen in other brain regions.

Slow-wave activity, a sign of deep sleep, had been linked to neuroplasticity in the visual cortex and sensorimotor cortex, according to a prior study (Bernardi et al., 2019). The sensorimotor cortex’s electroencephalogram recordings showed the presence of unique oscillating waves in the 12–15 Hz range, or sensorimotor rhythm (SMR). Improved sleep quality was a result of elevated SMR, which was connected to decreased nocturnal waking and greater slow-wave sleep in primary insomnia (Schabus et al., 2014). An earlier study using neuroimaging discovered that patients with chronic primary insomnia have a higher level of amplitude of low-frequency fluctuations (ALFF) in the posterior central gyrus/subparietal lobules, which is associated with poor sleep (Zhou et al., 2017). In comparison to healthy controls, patients with primary insomnia displayed higher ReHo in the cuneus (Wang et al., 2016). Acute sleep deprivation reduced the functional connection between the thalamus and the calcarine as well as the cuneus (Li et al., 2021). These findings suggest that the posterior central gyrus, calcarine, and cuneus are involved in sleep regulation.

According to a study using rs-fMRI, MCI patients had higher ReHo in the postcentral and paracentral lobes than people with normal cognitive performance. It was proposed that this rise might be a coping mechanism for the loss of frontal cognitive function (Wang et al., 2015). The ReHo of MCI patients with multiple cognitive domain impairments was lower than that of MCI patients with isolated memory deficits (Luo, et al., 2018). When compared to MCI patients without depressive symptoms, the ReHo of the left posterior central gyrus was lower in MCI patients with depressive symptoms, and this decrease was linked to the dysregulation of emotion processing (Liu et al., 2019). Compared with MCI patients without lacunar infarctions, MCI patients with lacunar infarctions had a lower ReHo in the precuneus/cuneus (Ni et al., 2016). In a recent study, AD patients with poor sleep showed reduced ALFF in the right cuneus compared with AD patients with normal sleep (Li et al., 2019). These results support our finding that the primary visual system (cuneus and calcarine) and the sensorimotor system (posterior central gyrus) perform compensatory roles in AD (Ferreri et al., 2003; Castellazzi et al., 2014). This modest compensatory effect, however, may appear as earlier decompensation when AD risk factors such as sleep disturbance, depression, vascular disease, and multi-domain cognitive impairment are combined. The cause of earlier decompensation in AD with sleep disturbances may be increased synaptic/neuronal activity in sleep-related brain regions brought on by sleep disorders. Increased synaptic/neuronal activity can result in an increase in the production, release, and decreased clearance of Aβ and tau in the brain’s interstitial fluid, which can impair synaptic/neuronal function, eliminate the compensatory effect, and finally worsen cognitive symptoms (Ju et al., 2014).

This study found decreased functional connectivity between the left posterior central gyrus and the left cuneus as well as the left calcarine, suggesting that functional connectivity between sensorimotor and visual networks is impaired in ADSD. Both the visual network (occipital lobe) and the sensorimotor network (anterior central gyrus, posterior central gyrus, and paracentral lobes) are related to visual-emotional deficits (Guo et al., 2012; Chen et al., 2019) and psychomotor agitation or retardation (Northoff, 2016). Stroke patients with impaired sensorimotor integration utilize the cross-modal plasticity of a visual network in motor function recovery (Seitz et al., 1999). It indicates that the two networks are in some sort of functional or compensatory interaction.

The posterior central gyrus belongs to the primary sensory cortex and is involved in sensorimotor integration (Claret et al., 2019). Sensorimotor integration is the ability of the central nervous system to integrate different stimulus sources (including somatosensory, auditory, visual, etc.) and convert these inputs into motor commands (Coynel et al., 2010). In the later stages of AD, implicit memory impairment leads to changes in motor control or motor ability that are associated with a lack of integration of sensory information into motor planning (Machado et al., 2009; Velasques et al., 2011). Typically, memory, visual, and sensory or motor impairments are considered features of aging, and patients with MCI perform poorly in these functions, while those with AD do much worse (Galton et al., 2000). Therefore, earlier research has come to the conclusion that one of the causes of early cognitive impairment in AD may be disrupted or diminished connections within or between sensorimotor networks, visual networks, and default mode networks (Cai, et al., 2017). Greater visual and somatosensory integration is also linked to better balance and a lower risk of falling as people age normally (Mahoney and Verghese, 2018). According to a longitudinal study using independent component analysis (ICA), patients with very mild AD had fewer functional connections between the visual and sensorimotor networks than normal controls, and it was thought that these fewer functional connections between the networks indicated the transition from the cognitively normal stage to the early stage of AD (Sendi et al., 2020). In conclusion, one of the factors accelerating the development of cognitive impairment in ADSD is the impaired functional connectivity between the sensorimotor network and the visual network brought on by sleep disturbances.

AD neuropathology has shown that neurofibrillary pathology associated with highly phosphorylated tau protein preferentially affects specific brain regions, and Aβ deposition also follows a certain spatial pattern (Thal et al., 2002; Braak et al., 2006). A study that combined rs-fMRI and ^18^F-AV 1451 tau-sensitive positron emission tomography (PET) has found that the spatial patterns of tau pathology primarily affect higher-order cognitive networks rather than primary sensorimotor networks. It overlaps with the dorsal attention network and, to some extent, with the higher-order vision, limbic, and default mode networks (Hansson et al., 2017). Another study used AV-45 PET, FDG-PET, and structural MRI to evaluate regional amyloid load, neuronal metabolism, and gray matter volume in the preclinical, predementia, and symptomatic AD stages. It was found that Aβ preferentially deposited in the default mode network, while its spatial distribution also overlapped considerably with the frontal parietal control network and the dorsal attention network, indicating that Aβ is similar to tau proteins and affects higher-order cognitive networks more generally (Grothe and Teipel, 2016). Additionally, task-based fMRI revealed that higher-order visual cortex and visual association cortex were preferentially involved in AD over primary visual cortex (Huang et al., 2020). This study found that ADSD had disrupted local and remote functional connections within or between the primary sensorimotor and primary visual networks. Higher-order cognitive networks were not affected, which may imply that sleep disturbances, a risk factor for AD, affect the temporal order of the brain functional networks that AD pathologically damages. Another explanation for such a finding is that network and connection specificity will be most evident earlier in the evolution of a particular disease (Warren et al., 2013). Unlike the mentioned studies focused on patients with MCI (Pini et al. and McKinnon et al.), the participants of the present study are patients with AD (the next stage in MCI development). In addition, functional interactions between large-scale brain networks will also tend to obscure network specificities (Chiong et al., 2013). However, caution should be used when interpreting this finding. More research, particularly the use of AD pathologically linked PET to determine whether the damaged brain area of ADSD also has a more severe pathological burden, is required to support this conclusion.

Our study still has certain limitations. First, rather than the self-reported questionnaire utilized in this study, objective tests like polysomnography could offer more thorough information on sleep disturbances. Second, rs-fMRI parameters are not that trustworthy due to the thresholds for correction for multiple comparisons. Remote functional connections may be affected by global signals or head motion, and the different choice of seed may produce different results. The relevant conclusions in this study were based on the promoting effect of sleep disorders on the pathology of AD. However, there were no indicators to evaluate the pathological burden of AD, which to some extent affected the reliability of rs-fMRI results. More research involving individuals with pathologically proven AD and a bigger sample size is required, particularly to determine whether the brain regions with altered functional connectivity have a significantly higher pathologic burden. Third, previous studies have shown that lacunar infarction affects the brain function of AD patients, suggesting that vascular damage is also a risk factor for AD (Ni et al., 2016). Although patients with multiple lacunar infarction, severe white matter lesions, and neck and intracranial vascular stenosis (>50%) were excluded in this study, vascular risk factors should be further controlled in future studies.

The current study demonstrated that, compared with ADNSD, ADSD is impaired in both local and remote functional connectivity, manifested as reduced functional connectivity involving the primary sensory network and the primary visual network. The abnormality of the above functional connectivity is one of the reasons why sleep disorders promote cognitive impairment in AD. Moreover, sleep disorders change the temporal sequence of AD pathological damage to brain functional networks, but more evidence is needed to support this conclusion. In future studies, evaluating the pathological burden of brain regions in ADSD is particularly important for analyzing the effects of sleep disorders on brain function in AD.

## Data availability statement

The original contributions presented in the study are included in the article/Supplementary material, further inquiries can be directed to the corresponding authors.

## Ethics statement

The studies involving humans were approved by China-Japan Friendship Hospital Ethics Committee. The studies were conducted in accordance with the local legislation and institutional requirements. Written informed consent for participation in this study was provided by the participants' legal guardians/next of kin.

## Author contributions

LW: Formal analysis, Writing – original draft, Writing – review & editing. RZ: Data curation, Writing – original draft. XZ: Data curation, Writing – original draft. ZZ: Methodology, Writing – review & editing. DP: Methodology, Writing – review & editing.

## Footnotes

^1^ https://www.fil.ion.ucl.ac.uk/spm/
