# Supplementary material for: Altered local and remote functional connectivity in mild Alzheimer’s disease patients with sleep disturbances
Source: Front Aging Neurosci. 2023 Oct 18;15:1269582. doi: 10.3389/fnagi.2023.1269582 (PMC10619161; doi:10.3389/fnagi.2023.1269582)
Supplement: Supplementary file 1 [file Data_Sheet_1.docx]

**
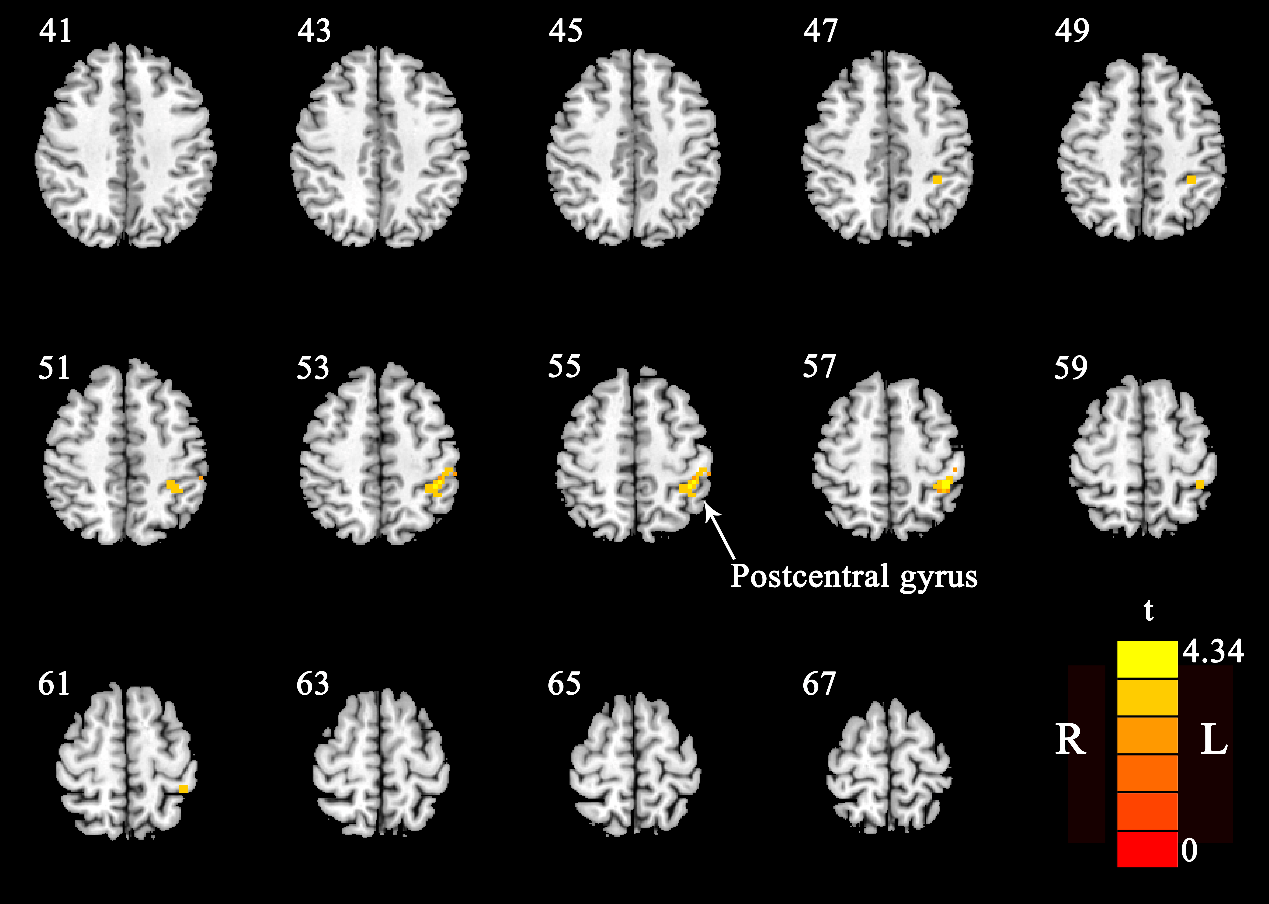
**

**Figure.s1:** Yellow represented increased dynamic ReHo in AD patients with sleep disturbances compared to AD patients without sleep disturbances (window width=60TR, step size=2TR; two-sample t-test, GRF corrected, voxel-wise p < 0.005, cluster-wise p < 0.05, two-tailed). R=right hemisphere; L=left hemisphere; the color bar represents the t value.


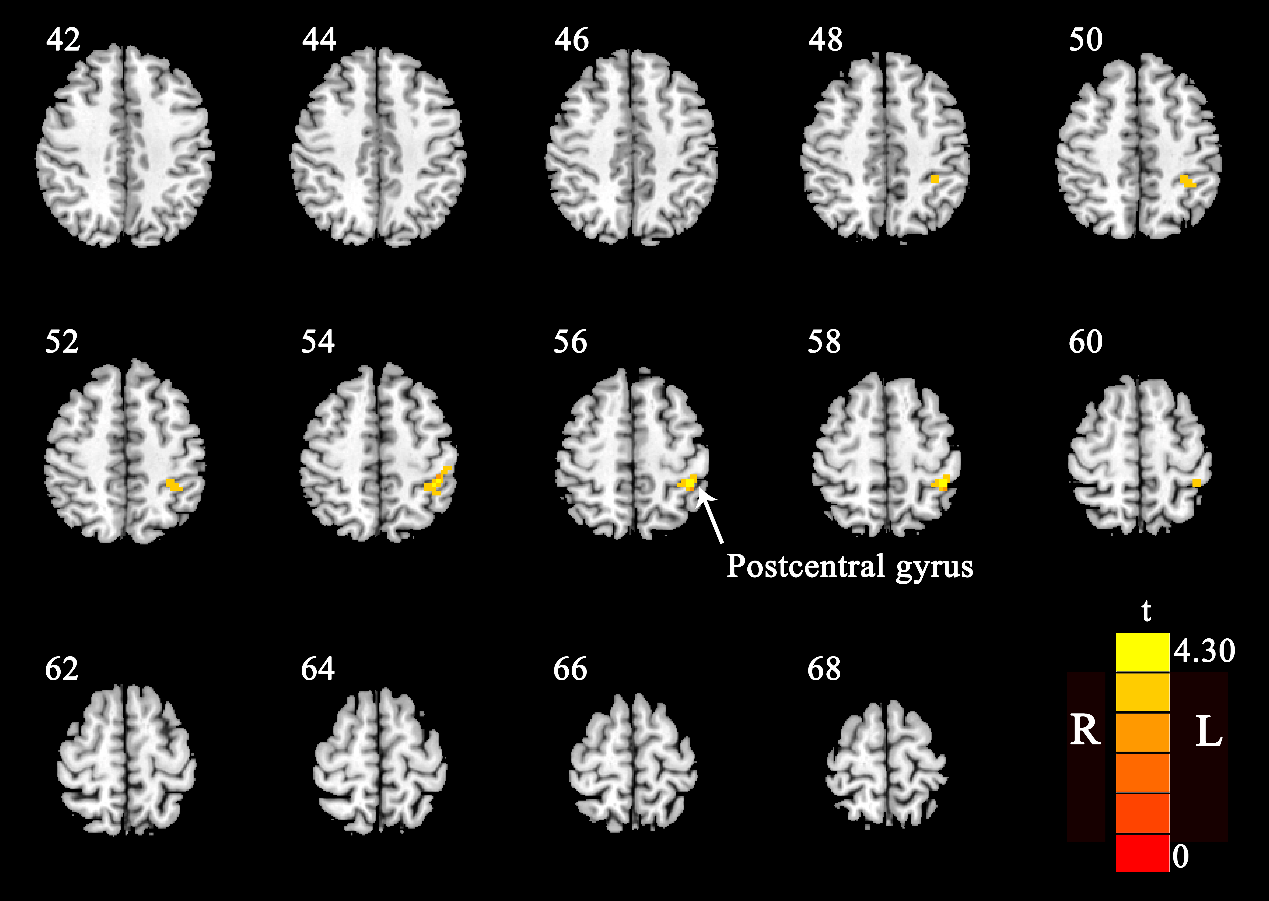


**Figure.s2:** Yellow represented increased dynamic ReHo in AD patients with sleep disturbances compared to AD patients without sleep disturbances (window width=60TR, step size=5TR; two-sample t-test, GRF corrected, voxel-wise p < 0.005, cluster-wise p < 0.05, two-tailed). R=right hemisphere; L=left hemisphere; the color bar represents the t value.


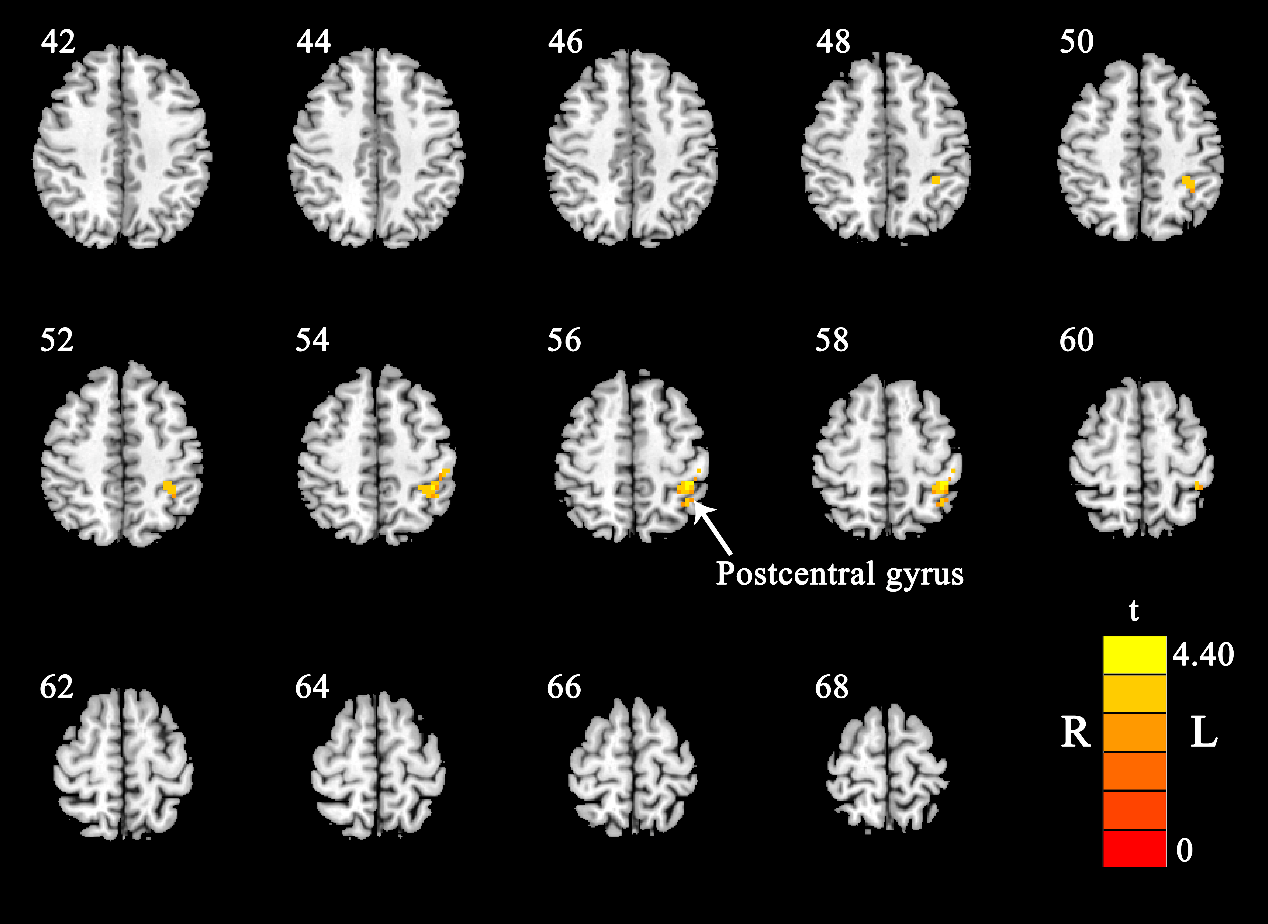


**Figure.s3:** Yellow represented increased dynamic ReHo in AD patients with sleep disturbances compared to AD patients without sleep disturbances (window width=75TR, step size=2TR; two-sample t-test, GRF corrected, voxel-wise p < 0.005, cluster-wise p < 0.05, two-tailed). R=right hemisphere; L=left hemisphere; The color bar represents the t value.

| Brain Regions | BA | Cluster Size (no.voxels) | Peak MNI coordinates | | | t value |
| --- | --- | --- | --- | --- | --- | --- |
|  |  |  | *X* | *Y* | *Z* |  |
| Increased dynamic ReHo in ADSD | | | | | | |
| Window width=60TR, step size=2TR | | | | | | |
| Left posterior central gyrus | 40 | 50 | -39 | -42 | 57 | 4.34 |
| Window width=60TR, step size=5TR | | | | | | |
| Left posterior central gyrus | 40 | 46 | -39 | -42 | 57 | 4.30 |
| Window width=75TR, step size=2TR | | | | | | |
| Left posterior central gyrus | 40 | 50 | -39 | -42 | 57 | 4.40 |

**Supplementary Table. Dynamic ReHo results in different window width and step size**

Notes: ADSD=AD patients with sleep disturbances; MNI=Montreal Neurological Institute; BA=Brodmann area.
